# Delayed Cellular Immunity in SARS-CoV-2 Antibody-Non-Responders to COVID-19 Vaccination: Rethinking Post-Vaccine Immune Assessment

**DOI:** 10.3390/vaccines13111123

**Published:** 2025-10-31

**Authors:** Dimitris Nikoloudis, Kanella E. Konstantinakou, Alexandros D. Konstantinidis, Natalia I. Spyrou, Irene V. Vasileiou, Athanasios Tsakris, Vassiliki C. Pitiriga

**Affiliations:** 1 Bioiatriki Healthcare Group, Kifisias 132 and Papada Street, 11526 Athens, Greece; dnicolgr@hotmail.com (D.N.);; 2 Department of Microbiology, Medical School of Athens, National and Kapodistrian University of Athens, 75 Mikras Asias Street, 11527 Athens, Greece; atsakris@med.uoa.gr

**Keywords:** COVID-19, SARS-CoV-2, vaccination, T-SPOT COVID, cellular immunity, T-cell immunity

## Abstract

Background: While host immune responses to SARS-CoV-2 vaccination are routinely assessed through IgG measurements, less is known about the temporal dynamics of vaccine-induced cellular immunity, particularly in individuals who fail to develop detectable IgG antibodies after COVID-19 vaccination. Objective: To investigate the development and timing of T-cell immunity following SARS-CoV-2 vaccination in antibody-non-responders to COVID-19 vaccination. Methods: A cross-sectional analysis was conducted on COVID-19-naive individuals who had received full SARS-CoV-2 vaccination, categorized by SARS-CoV-2 IgG serostatus. T-cell response was evaluated using the IGRA methodology T-SPOT^®^.COVID (Oxford Immunotec, Abingdon, Oxfordshire, UK). T-cell response rates and levels were compared between SARS-CoV-2 seropositive and seronegative groups, and a temporal cutoff analysis was applied to examine trends in T-cell response over time. Results: Within the seronegative group, IgG levels showed a strong negative correlation with time since vaccination (Spearman ρ = −0.65, *p* < 0.001), while T-cell response levels exhibited a weak positive time-dependent trend (ρ = 0.15, *p* = 0.019). Temporal cutoff analysis identified a critical time-point beginning at 80 days post-vaccination, after which both T-cell response rates and levels were significantly higher. Specifically, individuals tested after 80 days showed increased median T-cell response levels (U = 4205, *p* < 0.001) and higher positive T-cell response rate (67% vs. 38%, Χ^2^ = 17.06, *p* < 0.001). Conclusions: Cellular immunity response against SARS-CoV-2 may emerge later than expected in antibody-non-responders to COVID-19 vaccination, with the 80-day post-vaccination mark emerging as a critical time point. Our results support the inclusion of cellular assays in post-vaccination monitoring and emphasize the need to reconsider the timing and criteria for evaluating vaccine response.

## 1. Introduction

The global response to the COVID-19 pandemic has relied heavily on mass vaccination campaigns, most prominently with mRNA-based vaccines [1]. These vaccines have demonstrated robust efficacy in preventing severe disease and reducing transmission, primarily through the induction of both humoral and cellular immune responses [2,3]. However, real-world data and follow-up studies have increasingly highlighted the heterogeneity of immune responses among vaccinated individuals [4].

Immunological protection against SARS-CoV-2 involves two interrelated but distinct components: humoral immunity, primarily mediated by neutralizing antibodies such as IgG, and cellular immunity, which involves T-cell responses targeting viral antigens. While antibody titers are easily measurable and have become the standard marker for assessing post-vaccination immunity [5], they are known to decline over time, often dropping below detectable levels within months of vaccination [6]. In contrast, T-cell responses are typically more durable [7] and play a vital role in long-term immunity, particularly in reducing disease severity upon viral exposure.

Despite widespread recognition of this dual-arm immune architecture, current vaccination monitoring practices often neglect cellular immunity [8,9,10]. Notably, a negative antibody result is frequently used as the sole criterion for assessing immune protection or determining the need for additional vaccine doses, potentially misclassifying individuals who possess protective cellular responses [11].

Moreover, the kinetics of the T-cell response following vaccination remain less well defined, particularly in antibody non-responders after vaccination. While studies have shown that T-cell responses typically emerge within 1–2 weeks and up to one month post-vaccination in immunocompetent individuals [12], this timeline may differ in those with impaired or delayed humoral responses [13]. It remains unclear whether cellular immunity in these individuals is merely delayed, entirely absent, or uncoupled from the antibody response.

The present study addresses this knowledge gap by examining the development of cellular immunity in a cohort of vaccinated, COVID-19-naïve individuals who were tested for T-cell response using the ELISpot interferon-gamma release assay (IGRA) methodology. We specifically focused on individuals who tested negative for SARS-CoV-2-specific IgG after completing the first full vaccination scheme. Building upon our previous study [14], which showed that SARS-CoV-2-vaccinated individuals who were IgG seronegative exhibited lower levels of T-cell responses compared to seropositive individuals, this study aimed to compare T-cell responses at different time points post-vaccination to determine whether cellular responses in IgG-negative individuals follow a different kinetic pattern. Understanding these dynamics could enhance post-vaccination immune profiling, guide the optimal timing for follow-up testing, and inform decisions regarding booster administration—particularly in populations at risk of being misclassified based solely on antibody status.

## 2. Methods

### 2.1. Study Design and Participants

A retrospective, descriptive analysis was carried out using data obtained from electronic medical records of adult individuals who visited the “BIOIATRIKI” Healthcare Center—a major medical facility in Attica—between September 2021 and December 2022. These individuals sought SARS-CoV-2 immunity testing on their own initiative during the COVID-19 pandemic. The study included adults who were fully vaccinated and had no prior exposure to COVID-19.

Eligibility criteria required participants to have no history of COVID-19-related symptoms or known contact with confirmed cases, and no evidence of prior SARS-CoV-2 infection before vaccination, as documented by negative polymerase chain reaction (PCR), rapid antigen, or self-test results, together with negative IgG/IgM antibody assessments. Only individuals who had completed their primary vaccination regimen without receiving any booster doses at the time of testing were considered. Individuals with known immunocompromised status were excluded from the study.

The date marking the completion of primary vaccination (either the first or second dose, depending on vaccine type) was recorded as the individual’s vaccination date. The time elapsed between the last vaccine dose and sample collection was recorded in days and used as a variable in all time-based analyses. Additional demographic and medical history data were gathered through structured questionnaires routinely administered at the time of examination. Although the specific vaccine type administered was not documented for each participant, mRNA vaccines were widely available in Greece during the study period.

All blood samples were collected during the blood sampling hours (08:00 to 10:00). The participants were divided into two groups based on their SARS-CoV-2 IgG levels at the time of testing: those with a positive (≥50 AU/mL; ≥7 BAU/mL) and those with a negative IgG measurement (<50 AU/mL; <7 BAU/mL).

The study protocol received ethical approval from the institution’s review board on 29 June 2021 (6th Annual Meeting).

### 2.2. Laboratory Procedures

#### 2.2.1. ELISpot Assay for Detection of IFN-γ T-Cell Responses

Peripheral blood samples were collected into lithium heparin tubes during routine morning blood collection hours (08:00–10:00) and processed within 6 to 8 h of venipuncture, in accordance with the manufacturer’s guidelines.To evaluate T-cell-mediated immune response to SARS-CoV-2, the T-SPOT^®^.COVID test (Oxford Immunotec Global PLC, Abingdon (Milton Park), Oxfordshire, UK) was used. This assay, based on the ELISpot platform, detects T-cell responses by measuring the release of interferon-gamma (IFN-γ) in response to SARS-CoV-2 antigens, specifically spike (S) and nucleocapsid (N) proteins. Blood samples were collected into lithium heparin tubes during routine morning blood collection hours and processed within 6 to 8 h of venipuncture by treatment with T-Cell Xtend reagent. Peripheral blood mononuclear cells (PBMCs) were isolated, washed, counted, and plated into a 96-well plate at approximately 250,000 cells per well. Antigen peptide pools targeting the S and N proteins were added to two separate wells. A mitogen (phytohemagglutinin) was used as a positive control, while cell culture medium alone served as the negative control. After an incubation period of 16–20 h, wells were washed and treated with a conjugated secondary antibody that binds to IFN-γ. Unbound components were removed through additional washing, and a substrate was introduced to visualize IFN-γ secretion as dark spots, which were manually counted under a microscope by trained personnel. Results were assessed separately for responses to the S and N antigens. A test result was deemed invalid if the negative control exhibited more than 10 spot-forming cells (SFCs) or if the positive control yielded fewer than 20 SFCs and the antigen wells showed no reactivity. The predefined threshold for a positive response was 6 SFCs. A borderline range of ±1 SFC (i.e., 5–7 SFCs) was established to account for assay variability. Based on these criteria, results were classified as reactive (≥8 SFCs), non-reactive (≤4 SFCs), or borderline (5–7 SFCs) for the respective antigens.

#### 2.2.2. Measurement of SARS-CoV-2 IgG Antibodies

Blood samples were collected during routine morning blood collection hours, processed shortly after collection, and analyzed on the same day. Quantification of SARS-CoV-2-specific IgG antibodies was conducted using the SARS-CoV-2 IgG II Quant assay developed by Abbott Laboratories. This is a chemiluminescent microparticle immunoassay (CMIA) performed on the Alinityi system. It detects and quantifies IgG antibodies targeting the receptor binding domain (RBD) of the S1 subunit of the spike protein, using sequences from the WH-Human 1 coronavirus (GenBank accession MN908947). Blood serum samples were analyzed on the same day of examination. The assay reports results within an analytical range of 21 to 40,000 arbitrary units per milliliter (AU/mL; or 3 to 5714 BAU/mL), with a positivity threshold set at ≥50 AU/mL (≥7 BAU/mL), according to the manufacturer’s specifications.

### 2.3. Statistical Analysis

All possible pairwise correlations were assessed using either the Spearman ρ coefficient for continuous variables or the Point biserial r coefficient between continuous and dichotomous variables (i.e., sex).

We subsequently performed a cutoff analysis across all distinct post-vaccination time points in our dataset (104 cutoffs ranging from 14 to 364 days), conducted separately within the IgG-negative and IgG-positive cohorts. At each cutoff, participants were dichotomized into “early” (≤cutoff) and “late” (>cutoff) groups. For the continuous S-antigen response levels we applied the Wilcoxon rank-sum (U) test, and for binary S-antigen positivity outcomes we used Pearson’s chi-square test (without Yates correction). This exploratory scan identified a region of particular interest between 59 and 169 days post-vaccination. To rigorously assess statistical significance within this window while accounting for multiple testing, we conducted a permutation-based adjustment using the 40 distinct post-vaccination time points available in the dataset between 59 and 169 days. In each of 100,000 permutations, we randomly shuffled the S-antigen positivity labels among participants, re-ran the series of 40 chi-square tests, and recorded the minimum *p*-value observed in that permutation. The permutation-adjusted *p*-value was defined as the proportion of permutations in which the permuted minimum *p*-value was equal to or smaller than the observed minimum *p*-value.

Wilcoxon-Mann-Whitney U tests were also employed to compare the medians of various parameters, between the seronegative and seropositive groups. All statistical analyses were performed in R (version 4.4.0). Parallel computation was implemented via the ‘parallel’ package to expedite the 100,000 permutations. Throughout the analysis, two-sided *p*-values < 0.05 were considered statistically significant.

## 3. Results

The study involved a total of 289 participants, including 163 women (56.4%) and 126 men (43.6%), with ages ranging from 17 to 92 years (mean age: 59.10 ± 15.3 years). Detailed demographic and clinical data of the participants are summarized in Table 1. T-SPOT results for the N antigen were negative (SFC ≤ 4) for all participants. Individuals were divided into two groups: those with a positive SARS-CoV-2 IgG result (≥50 AU/mL; ≥7 BAU/mL) on the day of testing and those with a negative result (<50 AU/mL; <7 BAU/mL).

**Figure 1:**
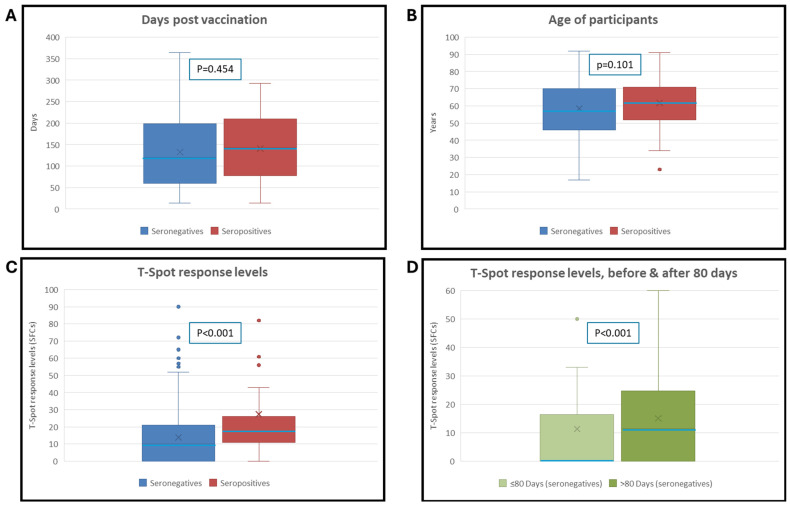
Whisker plots comparing the distributions between SARS-CoV-2 IgG seronegative and seropositive groups for: (**A**) days elapsed since vaccination (U test, *p* = 0.454), (**B**) participant age (U test, *p* = 0.101), (**C**) T-SPOT response levels to S antigen (U test, *p* < 0.001), and (**D**) Comparison of T-SPOT response levels to S antigen in seronegative individuals before and after 80 days post-vaccination (U test, *p* < 0.001).

Both T-SPOT positivity rates for the S antigen and levels differed significantly between the two groups (U test, *p* < 0.001) (Table 2; Figure 1A,C). Days since vaccination, sex, and age did not differ significantly between the groups.

In the SARS-CoV-2 IgG-negative group, detailed pairwise plots revealed a strong negative correlation between IgG levels and days since vaccination (Spearman ρ = −0.65, *p* < 0.001). Furthermore, a weak correlation was observed in the seronegative group between T-SPOT measurements and days since vaccination (Spearman ρ = 0.15, *p* = 0.019). The same effect was not observed in the seropositive group (ρ = 0.07, *p* = 0.58).

**Figure 2:**
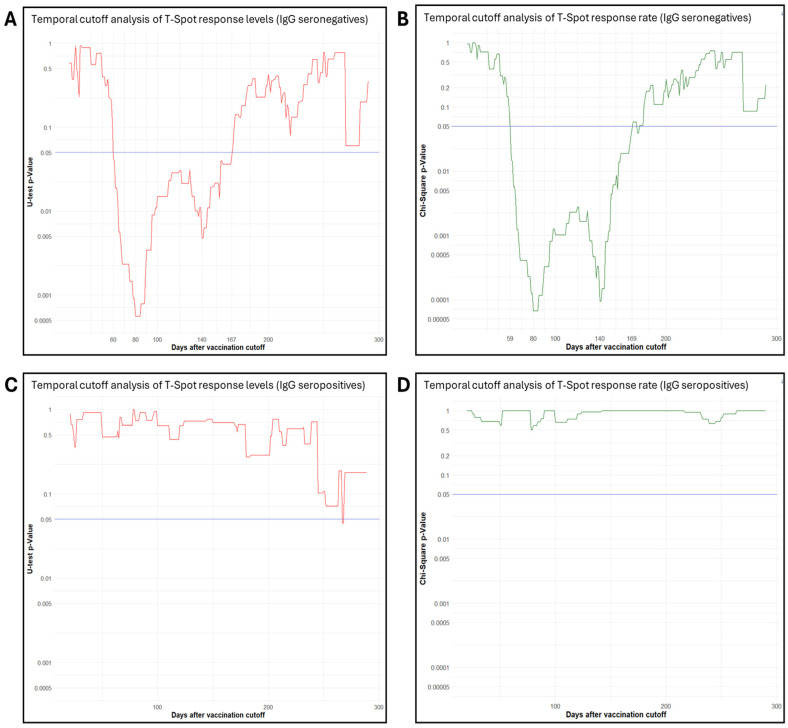
Cutoff analysis of the temporal impact on T-SPOT levels and the development of cellular immunity using 1-day steps. Traces represent *p*-values from consecutive statistical tests of subgroups formed before and after successive time cutoffs; the blue line indicates the conventional threshold for statistical significance (*p* = 0.05): (**A**) U tests for T-SPOT response levels to S antigen in the SARS-CoV-2 IgG-seronegative group, (**B**) chi-square tests for T-SPOT positive response rate to S antigen in the IgG-seronegative group, (**C**) U tests for T-SPOT response levels to S antigen in the SARS-CoV-2 IgG-seropositive group, and (**D**) chi-square tests for T-SPOT positive response rate to S antigen in the SARS-CoV-2 IgG-seropositive group.

To investigate the temporal impact on the development of cellular immunity and T-SPOT measurements for S antigen in seronegative individuals after vaccination, we conducted a comprehensive cutoff analysis using 1-day intervals. This analysis involved statistical comparisons of T-SPOT measurements and positivity rates for S antigen on either side of each cutoff point, tracking *p*-value trends (Figure 2). In the IgG-negative group, consecutive U tests revealed a significant difference in T-SPOT measurements for S antigen between subgroups formed before and after time cutoffs between 60 and 167 days. Within this window, the “late” subgroup (i.e., individuals tested after each cutoff) consistently exhibited significantly higher T-SPOT measurements. The strongest statistical significance (i.e., the lowest *p*-value) was observed at 80 days (U test, *p* < 0.001), suggesting a potential critical time point for the development of cellular immunity. A second distinctive peak in the window of statistical significance was observed at 140 days (U test, *p* = 0.047) (Figure 2A).

For T-SPOT positivity rates for S antigen in the IgG-negative group, consecutive chi-square tests revealed a similar window of significance between 59 and 169 days, with two distinct *p*-value minima observed at 80 days (Χ^2^ (1), N = 230, *p* < 0.001) and 140 days (Χ^2^ (1), N = 230, *p* < 0.001) (Figure 2A). At all-time points within this range, the subgroup formed after each cutoff consistently exhibited a significantly higher positivity rate. Permutation analysis for the 59–169-day window, yielded an adjusted *p*-value of *p* < 0.001. Given this strong validation result, both observed cutoffs were retained as significant time points for post-vaccination T-SPOT positivity for S antigen in the IgG-negative group. Beyond the period of statistical significance, both seronegative and seropositive individuals with detectable cellular immunity at or after 170 days post-vaccination (44 seronegatives vs. 21 seropositives) exhibited comparable median T-SPOT values of 18 and 20 SFCs, respectively (U test, *p* = 0.71). In the IgG-positive group, the same cutoff analysis did not identify any significant time points. (Figure 2C,D).

Table 3 demonstrates the comparison of individuals tested before and after 80 days post-vaccination among SARS-CoV-2 IgG-seronegative participants.

## 4. Discussion

In our study, we observed that individuals who did not develop detectable antibodies after COVID-19 vaccination exhibited a delayed onset of cellular immune responses compared to individuals who developed antibodies. As all individuals had no documented history of SARS-CoV-2 infection prior to vaccination, all measurements for the N antigen were negative (SFC ≤ 4), confirming the absence of previous natural infection. Therefore, the observed T-cell reactivity in this cohort reflects responses specifically directed against the Spike antigen, consistent with vaccine-induced immunity.

Notably, T-cell–positive response levels to S antigen in IgG-negative participants tested within 60–167 days post-vaccination were significantly lower than those tested later, with the most pronounced difference observed at the 80-day cutoff, beyond which T-cell–positive responses increased significantly.

Similarly, our cutoff analysis of T-SPOT positivity rates in the IgG-negative group revealed a continuous window of statistical significance between 59 and 169 days post-vaccination, with distinct *p*-value minima at 80 and 140 days. We interpret the 80-day minimum as an inflection point beyond which the rate of positive T-cell responses increases markedly. In contrast, the second significant *p*-value minimum at 140 days may reflect the higher T-SPOT positivity rate among individuals who transitioned from the seropositive to the seronegative group due to antibody waning.

Interestingly, beyond the end of the identified window of statistical significance, both groups of IgG seronegatives and seropositives that have developed T cell immunity, became very similar in their T-SPOT measurements. This observation suggests that once a robust T-cell response is established, its magnitude remains relatively stable over time. This pattern of sustained cellular immunity is consistent with longitudinal studies showing that T-cell responses to SARS-CoV-2 remain durable for at least six months post-vaccination, even in the presence of significant declines in antibody titers [15].

In contrast, T-cell responses for S antigen in the IgG-positive group appeared to be established early, consistent with previous reports indicating that robust cellular immunity can develop as early as 1–2 weeks after completing a two-dose vaccination regimen [16,17,18], and typically within one month [19,20]. Notably, the IgG-negative group was larger (230 out of 289 participants) than the IgG-positive group, since a negative humoral immunity result was the main reason for ordering a follow-up T-cell immunity test—to assess whether vaccination had successfully elicited at least one form of SARS-CoV-2–specific immune response.

The percentage of IgG-seropositive individuals who also demonstrated a positive T-cell response to the S antigen was 88%, somewhat lower than the 95% reported in participants of the Pfizer-BioNTech BNT162b1 vaccine trials [21]. The study population included only individuals aged 18–55 years, whereas in our cohort, most seropositive participants lacking a T-cell response were older (59–85 years). This age difference likely accounts for the lower proportion of individuals exhibiting both humoral and cellular immunity observed in our cohort.

Several mechanisms may underlie this delayed development in the IgG-negative group according to current literature. Firstly, it is possible that a subset of individuals who fail to mount a humoral response initially—due to factors such as underlying B-cell dysfunction or other immunological constraints [22,23,24]—also experience a slower maturation of cellular immunity. This finding aligns with literature on immunocompromised patients, where robust SARS-CoV-2–specific T-cell responses for S antigen are detected only several weeks after vaccination [25].

Furthermore, intrinsic variability in immune responsiveness—shaped by genetic polymorphisms affecting antigen presentation, T-cell receptor diversity, and cytokine signaling—can influence the efficiency of cellular immunity [26]. Age-related immunosenescence may also contribute, even in the absence of overt immunosuppression, by impairing both humoral and cellular responses [27]. In addition, host genetic background (including HLA type), epigenetic modifications [28,29], and variability in vaccine uptake or antigen expression can further affect the timing and magnitude of T-cell responses [30].

On the other hand, while individuals who initially develop protective levels of IgG are most likely to be classified into the SARS-CoV-2 IgG-positive group, those tested at later intervals (e.g., after 4 months post-vaccination) may be falsely classified as IgG negative due to a significant drop in IgG levels by the time of testing. This interpretation is supported by systematic reviews indicating that although antibody levels may diminish by over 75% at five months post-vaccination [31], T-cell responses tend to be more stable [32,33]. Therefore, their classification as IgG negative may be contributing to the observation of an increased T-cell response rate in the later time window.

This finding of the 80-day time point suggests that assessing SARS-CoV-2 T-cell responses beyond 80 days post-vaccination may offer a more accurate evaluation of vaccine-induced immunity and could inform the optimal timing for post-vaccination immune assessment. However, the use of T-cell responses as a reference for determining the precise timing of booster immunization remains investigational. Although T-SPOT measurement levels may reflect sustained cellular immunity—particularly in individuals with undetectable IgG titers—standardized thresholds linking T-SPOT results to clinical protection are currently lacking. Thus, T-SPOT levels can serve as a supportive biomarker for assessing ongoing cellular immunity and informing the design of booster vaccination schedules, especially in populations at increased risk of waning immunity.

A key strength of this study is its focus on a well-defined cohort: COVID-19-naïve individuals who completed a primary immunization schedule, predominantly two doses of the Pfizer-BioNTech mRNA vaccine. By excluding previously infected or boosted participants, the study provides a clearer assessment of cellular immunity induced by primary vaccination alone, enabling a more accurate examination of the natural trajectory of T-cell responses in seronegative individuals.

Certain limitations of the study should also be acknowledged. First, the cross-sectional design of the study captures immune responses at a single time point per individual, which limits our ability to track the true kinetics of humoral and cellular immunity over time. Although we attempted to address this limitation by analyzing aggregated trends across different time intervals, future studies involving serial sampling from the same individuals will be necessary to confirm true longitudinal patterns. Moreover, no additional data, such as the type of the administered vaccines—except that the vast majority were mRNA—were available for further analysis.

The dataset was divided into IgG-seronegative and IgG-seropositive groups using the assay’s predefined threshold, rather than higher protective cutoffs (e.g., >899 BAU/mL in the ChAdOx1 nCoV-19 UK trial or >980 BAU/mL for Delta protection in dialysis patients) [34,35]. This choice reflected a key feature of our cohort: all participants had both IgG quantification and T-SPOT testing, enabling concurrent evaluation of humoral and cellular immunity. As only 8 of 262 individuals exceeded these protective thresholds, applying higher cutoffs would not have been statistically meaningful and was therefore not used. Another limitation of this study was that despite the exclusion of immunocompromised individuals, which helped minimize major confounding effects on vaccine-induced immune despite this exclusion, other comorbidities—such as hypertension, diabetes, and cardiovascular disease—were present among participants. However, subgroup sizes were insufficient for robust multivariable modelling. Given the exclusion of immunosuppressed individuals and the lack of statistically significant differences in comorbidities between the 2 groups, we believe the main findings regarding T-cell and IgG responses remain valid and interpretable. Finally, the classification of individuals as IgG-negative may have included those who had previously mounted an antibody response but experienced rapid waning, introducing heterogeneity into this group.

## 5. Conclusions

In summary, our cross-sectional studysuggests that T-cell responses in IgG-negative individuals are more frequently observed at later time points after vaccination against SARS-CoV-2. Assessing cellular immunity is therefore critical for understanding protection against SARS-CoV-2, particularly in those who fail to develop detectable antibody responses. These findings support the integration of SARS-CoV-2–specific T-cell assays into post-vaccination monitoring and underscore the need to refine both the timing and the criteria used to evaluate vaccine responses. Further longitudinal studies are needed to confirm and extend these observations.

## Figures and Tables

**Table 1:** Clinical and demographic characteristics of participants in the study groups.

Variables	IgG Negatives (N = 230)	IgG Positives (N = 59)	*p*-Value
**Demographic Characteristics**			
Sex (F/M)	131/99	32/27	NS
Age (years ± SD)	58.4 ± 15.5	61.8 ± 14.1	NS
Comorbidities			
Respiratory disorders	33 (14.3)	4 (6.7)	NS
Cardiovascular diseases	30 (13.0)	5 (8.4)	NS
Central nervous system disorders	1 (0.4)	0	NS
Diabetes mellitus	13 (5.6)	4 (6.7)	NS
Hypertension	30 (13.0)	10 (16.9)	NS
Lipidemia	52 (22.6)	9 (15.2)	NS
Obesity	28 (12.1)	10 (16.9)	NS

Abbreviations: F/M—Female/Male; NS—Not Significant; SD—Standard Deviation; N-Number.

**Table 2:** Comparison between groups of SARS-CoV-2 IgG seronegatives and seropositives.

	IgG Negatives (N = 230)	IgG Positives (N = 59)	Statistical Test	*p*-Value
T-SPOT% positivity rate for S antigen	57% (132/230)	88% (52/59)	Χ^2^ = 19.187	<0.001
T-SPOT MEDIAN (No of SFC)	10	17	Mann-Whitney U = 9005	<0.001
T-SPOT MEAN (No of SFC) for S antigen	14	27		
Days after vaccination MEDIAN	120	141	Mann-Whitney U = 7214	0.454
Days after vaccination MEAN	132	142		
Sex (% Female)	57% (131/230)	54% (32/59)	Χ^2^ = 0.141	0.713
Age MEDIAN (years)	57	62	Mann-Whitney U = 7724	0.101
Age MEAN (years)	58	62		

Abbreviations: No: Number; SFC: spot-forming cells.

**Table 3:** Comparison between individuals tested before and after 80 days post-vaccination among SARS-CoV-2 IgG–seronegative participants.

	≤80 Days	>80 Days	Test Performed	*p*-Value
No. of samples (Total: 230)	74	156		
T-SPOT% positivity rate	38% (28/74)	67% (104/156)	Χ^2^ = 17.06	*p* < 0.001
T-SPOT MEDIAN (No of SFC)	0	12	Mann-Whitney U = 4205	*p* < 0.001
T-SPOT MEAN (No of SFC)	11	15		
Sex (% Female)	61% (45/74)	55% (86/156)	Χ^2^ = 0.66	0.423
Age MEDIAN (years)	54	59	Mann-Whitney U = 5274	0.291
Age MEAN (years)	57	59		

Abbreviations: No.: Number; SFC: spot-forming cells.

## Data Availability

All data from this study are included in this article.

## References

[1] Killeen T., Kermer V., Troxler Saxer R. (2023). mRNA Vaccine Development during the COVID-19 Pandemic: A Retrospective Review from the Perspective of the Swiss Affiliate of a Global Biopharmaceutical Company. J. Pharm. Policy Pract..

[2] Szabó G.T., Mahiny A.J., Vlatkovic I. (2022). COVID-19 mRNA vaccines: Platforms and current developments. Mol. Ther..

[3] Mirtaleb M.S., Falak R., Heshmatnia J., Bakhshandeh B., Taheri R.A., Soleimanjahi H., Emameh R.Z. (2023). An insight overview on COVID-19 mRNA vaccines: Advantageous, pharmacology, mechanism of action, and prospective considerations. Int. Immunopharmacol..

[4] Ciabattini A., Pettini E., Fiorino F., Polvere J., Lucchesi S., Coppola C., Costagli S., Pastore G., Sicuranza A., Tozzi M. (2025). Longitudinal immunogenicity cohort study of SARS-CoV-2 mRNA vaccines across individuals with different immunocompromising conditions: Heterogeneity in the immune response and crucial role of Omicron-adapted booster doses. EBioMedicine.

[5] Dhawan M., Thakur N., Sharma M., Rabaan A.A. (2025). The comprehensive insights into the B-cells-mediated immune response against COVID-19 infection amid the ongoing evolution of SARS-CoV-2. Biomed. Pharmacother..

[6] Reekie J., Stovring H., Nielsen H., Johansen I.S., Benfield T., Wiese L., Stærke N.B., Iversen K., Mustafa A.B., Petersen K.T. (2024). Development of antibody levels and subsequent decline in individuals with vaccine induced and hybrid immunity to SARS-CoV-2. Int. J. Infect. Dis..

[7] Pitiriga V.C., Papamentzelopoulou M., Konstantinakou K.E., Vasileiou I.V., Konstantinidis A.D., Spyrou N.I., Tsakris A. (2024). Prolonged SARS-CoV-2 T cell Responses in a Vaccinated COVID-19-Naive Population. Vaccines.

[8] Erra L., Uriarte I., Colado A., Paolini M.V., Seminario G., Fernández J.B., Tau L., Bernatowiez J., Moreira I., Vishnopolska S. (2022). COVID-19 Vaccination Responses with Different Vaccine Platforms in Patients with Inborn Errors of Immunity. J. Clin. Immunol..

[9] Yang Z.R., Jiang Y.W., Li F.X., Liu D., Lin T.F., Zhao Z.Y., Wei C., Jin Q.Y., Li X.M., Jia Y.X. (2023). Efficacy of SARS-CoV-2 vaccines and the dose–response relationship with three major antibodies: A systematic review and meta-analysis of randomised controlled trials. Lancet Microbe.

[10] Liu C., Tsang T.K., Sullivan S.G., Cowling B.J., Yang B. (2025). Comparative duration of neutralizing responses and protections of COVID-19 vaccination and correlates of protection. Nat. Commun..

[11] Pitiriga V.C., Papamentzelopoulou M., Konstantinakou K.E., Theodoridou K., Vasileiou I.V., Tsakris A. (2023). SARS-CoV-2 T-cell Immunity Responses following Natural Infection and Vaccination. Vaccines.

[12] Folegatti P.M., Ewer K.J., Aley P.K., Angus B., Becker S., Belij-Rammerstorfer S., Bellamy D., Bibi S., Bittaye M., Clutterbuck E.A. (2020). Safety and immunogenicity of the ChAdOx1 nCoV-19 vaccine against SARS-CoV-2: A preliminary report of a phase 1/2, single-blind, randomised controlled trial. Lancet.

[13] Paniskaki K., Anft M., Meister T.L., Marheinecke C., Pfaender S., Skrzypczyk S., Seibert F.S., Thieme C.J., Konik M.J., Dolff S. (2022). Immune Response in Moderate to Critical Breakthrough COVID-19 Infection After mRNA Vaccination. Front. Immunol..

[14] Pitiriga V.C., Papamentzelopoulou M., Nikoloudis D., Saldari C., Konstantinakou K.E., Vasileiou I.V., Tsakris A. (2025). Evaluating SARS-CoV-2 T Cell Immunity in COVID-19-Naive Vaccinated Individuals with and Without Spike Protein IgG Antibodies. Pathogens.

[15] Zuo J., Dowell A.C., Pearce H., Verma K., Long H.M., Begum J., Aiano F., Amin-Chowdhury Z., Hoschler K., Brooks T. (2021). Robust SARS-CoV-2-Specific T Cell Immunity Is Maintained at 6 Months Following Primary Infection. Nat. Immunol..

[16] Anderson E.J., Rouphael N.G., Widge A.T., Jackson L.A., Roberts P.C., Makhene M., Chappell J.D., Denison M.R., Stevens L.J., Pruijssers A.J. (2020). Safety and Immunogenicity of SARS-CoV-2 mRNA-1273 Vaccine in Older Adults. N. Engl. J. Med..

[17] Sahin U., Muik A., Derhovanessian E., Vogler I., Kranz L.M., Vormehr M., Baum A., Pascal K., Quandt J., Maurus D. (2020). COVID-19 vaccine BNT162b1 elicits human antibody and TH1 T-cell responses. Nature.

[18] Takeuchi J.S., Fukunaga A., Yamamoto S., Tanaka A., Matsuda K., Kimura M., Kamikawa A., Kito Y., Maeda K., Ueda G. (2022). SARS-CoV-2 specific T cell and humoral immune responses upon vaccination with BNT162b2: A 9 months longitudinal study. Sci. Rep..

[19] Tarke A., Coelho C.H., Zhang Z., Dan J.M., Yu E.D., Methot N., Bloom N.I., Goodwin B., Phillips E., Mallal S. (2022). SARS-CoV-2 vaccination induces immunological T cell memory able to cross-recognize variants from Alpha to Omicron. Cell.

[20] GeurtsvanKessel C.H., Geers D., Schmitz K.S., Mykytyn A.Z., Lamers M.M., Bogers S., Scherbeijn S., Gommers L., Sablerolles R.S.G., Nieuwkoop N.N. (2022). Divergent SARS-CoV-2 Omicron–reactive T and B cell responses in COVID-19 vaccine recipients. Sci. Immunol..

[21] Gallais F., Velay A., Nazon C., Wendling M.J., Partisani M., Sibilia J., Candon S., Fafi-Kremer S. (2021). Intrafamilial Exposure to SARS-CoV-2 Associated with Cellular Immune Response without Seroconversion, France. Emerg. Infect. Dis..

[22] Bacher P., Rosati E., Esser D., Martini G.R., Saggau C., Schiminsky E., Dargvainiene J., Schröder I., Wieters I., Khodamoradi Y. (2020). Low-Avidity CD4+ T Cell Responses to SARS-CoV-2 in Unexposed Individuals and Humans with Severe COVID-19. Immunity.

[23] Marasco V., Carniti C., Guidetti A., Farina L., Magni M., Miceli R., Calabretta L., Verderio P., Ljevar S., Serpenti F. (2022). T-cell immune response after mRNA SARS-CoV-2 vaccines is frequently detected also in the absence of seroconversion in patients with lymphoid malignancies. Br. J. Haematol..

[24] Jay C., Ratcliff J., Turtle L., Goulder P., Klenerman P. (2023). Exposed seronegative: Cellular immune responses to SARS-CoV-2 in the absence of seroconversion. Front. Immunol..

[25] Apostolidis S.A., Kakara M., Painter M.M., Goel R.R., Mathew D., Lenzi K., Rezk A., Patterson K.R., Espinoza D.A., Kadri J.C. (2021). Cellular and humoral immune responses following SARS-CoV-2 mRNA vaccination in patients with multiple sclerosis on anti-CD20 therapy. Nat. Med..

[26] Hoang Nguyen K.H., Le N.V., Nguyen P.H., Nguyen H.H.T., Hoang D.M., Huynh C.D. (2025). Human immune system: Exploring diversity across individuals and populations. Heliyon.

[27] Schwarz T., Tober-Lau P., Hillus D., Helbig E.T., Lippert L.J., Thibeault C., Koch W., Landgraf I., Michel J., Bergfeld L. (2021). Delayed Antibody and T-Cell Response to BNT162b2 Vaccination in the Elderly, Germany. Emerg. Infect. Dis..

[28] COVID-19 Host Genetics Initiative (2020). The COVID-19 Host Genetics Initiative, a global initiative to elucidate the role of host genetic factors in susceptibility and severity of the SARS-CoV-2 virus pandemic. Eur. J. Hum. Genet..

[29] Shi Y., Lu Y., You J. (2022). Antigen transfer and its effect on vaccine-induced immune amplification and tolerance. Theranostics.

[30] Pathak G.A., Singh K., Miller-Fleming T.W., Wendt F.R., Ehsan N., Hou K., Johnson R., Lu Z., Gopalan S., Yengo L. (2021). Integrative genomic analyses identify susceptibility genes underlying COVID-19 hospitalization. Nat. Commun..

[31] Notarte K.I., Guerrero-Arguero I., Velasco J.V., Ver A.T., Santos de Oliveira M.H., Catahay J.A., Khan S.R., Pastrana A., Juszczyk G., Torrelles J.B. (2022). Characterization of the significant decline in humoral immune response six months post-SARS-CoV-2 mRNA vaccination: A systematic review. J. Med. Virol..

[32] Goel R.R., Painter M.M., Apostolidis S.A., Mathew D., Meng W., Rosenfeld A.M., Lundgreen K.A., Reynaldi A., Khoury D.S., Pattekar A. (2021). mRNA vaccines induce durable immune memory to SARS-CoV-2 and variants of concern. Science.

[33] Tarke A., Sidney J., Methot N., Yu E.D., Zhang Y., Dan J.M., Goodwin B., Rubiro P., Sutherland A., Wang E. (2021). Impact of SARS-CoV-2 variants on the total CD4+ and CD8+ T cell reactivity in infected or vaccinated individuals. Cell Rep. Med..

[34] Feng S., Phillips D.J., White T., Sayal H., Aley P.K., Bibi S., Dold C., Fuskova M., Gilbert S.C., Hirsch I. (2021). Correlates of protection against symptomatic and asymptomatic SARS-CoV-2 infection. Nat. Med..

[35] Rostoker G., Rouanet S., Merzoug M., Chakaroun H., Griuncelli M., Loridon C., Boulahia G., Gagnon L. (2025). Serological Correlate of Protection Established by Neutralizing Antibodies Differs Among Dialysis Patients with SARS-CoV-2 Variants of Concern. Vaccines.

